# Phylogenetic analysis of a 2024 Sheeppox virus isolate from the Almaty region of Kazakhstan and investigation of its pathogenicity in merino sheep

**DOI:** 10.3389/fvets.2025.1623187

**Published:** 2025-07-21

**Authors:** Moldir Azanbekova, Muratbay Mambetaliyev, Aisulu Valiyeva, Nurlan Kozhabergenov, Nurbek Aldayarov, Sanat Kilibayev, Bekbolat Usserbayev, Moldir Tuyskanova, Balziya Kadyrova, Balzhan Myrzakhmetova, Lespek Kutumbetov, Olga Chervyakova, Sergazy Nurabayev, Maxat Berdikulov, Aslan Kerimbayev, Aralbek Rsaliyev, Yergali Abduraimov, Kuandyk Zhugunissov

**Affiliations:** ^1^Research Institute for Biological Safety Problems, National Holding “QazBioPharm”, Guardeyskiy, Kazakhstan; ^2^Kyrgyz-Turkish Manas University, Bishkek, Kyrgyzstan; ^3^National Reference Center for Veterinary, Astana, Kazakhstan; ^4^National Holding “QazBioPharm”, Astana, Kazakhstan

**Keywords:** Sheeppox, sequencing, phylogenetic analysis, virus, pathogenicity, pathological examination, histopathological changes

## Abstract

**Introduction:**

Sheeppox virus (SPPV) is a significant pathogen that affects small ruminants and causes substantial economic losses. It is essential to perform detailed molecular and pathogenic characterization of field isolates to control the disease and develop prevention strategies.

**Methods:**

The SPPV isolate “Sheeppox/KZ/Targap/2024” was obtained in 2024 from a diseased lamb during an outbreak in Targap village in the Almaty region of Kazakhstan. The isolate was passaged three times in lamb kidney-cell culture, and the 50% tissue culture infectious dose (TCID_50_) assay indicated a titer of 5.33 ± 0.08 log_10_ TCID_50_/mL. Identification was performed using polymerase chain reaction (PCR) and whole-genome sequencing, and the complete genome was submitted to GenBank (accession number PV434148). Experimental infection studies were conducted with Merino sheep.

**Results:**

The virus activity was 4.75 ± 0.02 log_10_ ID_50_/mL. The virus neutralization test showed 100% seroconversion by day 9 post-infection and maximum antibody titers (1:32) by day 21. ELISA confirmed that there was a strong immune response (>90% seropositivity). PCR detected viral DNA by day 5 post-infection in most tissues. Necropsy revealed typical pathological sheeppox lesions in the lungs, spleen, lymph nodes, and skin. Histopathological analysis demonstrated acute-stage features including massive cellular infiltration, vasculitis, edema, and pox lesions.

**Conclusion:**

The findings confirm previously known characteristics of SPPV and provide new insights into the molecular and pathogenic properties of the “Sheeppox/KZ/Targap/2024” isolate.

## 1 Introduction

Sheeppox is a highly contagious and economically significant viral disease that affects small ruminants, particularly sheep. The disease is characterized by papular and pustular skin lesions (exanthema), which are often accompanied by mucosal involvement. The causative agent is sheeppox virus (SPPV), which is a DNA-containing virus with a complex structure in the genus *Capripoxvirus* and the family Poxviridae. Morphologically, SPPV appears as a large oval or brick-shaped virion enveloped in a two-layered membrane (1, 2). Its genome comprises approximately 150 kilobase pairs (kb) of double-stranded linear DNA, which encode about 147–150 proteins that are involved in viral replication, immune evasion, and pathogenesis (3).

Transmission of SPPV occurs primarily through nasal secretions via airborne droplets, although alimentary transmission can also occur through contaminated feed and direct contact with infected material. Major sources of infection include manure, animal care equipment, and contaminated bedding (4–7). Fine-wool sheep breeds are particularly vulnerable to severe clinical manifestations of the disease (8). Clinically, infected animals display signs such as emaciation, hemorrhages, edema, vasculitis, and full-thickness necrosis of the skin. Pox lesions are also commonly found on the mucous membranes of the eyes, respiratory and gastrointestinal tracts, nostrils, external genitalia, and udders. In severe cases, lesions coalesce into extensive necrotic areas.

Pulmonary involvement is frequently observed and is characterized by diffuse pox lesions across the lung surface, hyperemia, edema, focal necrosis, and lobular atelectasis. The mediastinal lymph nodes often exhibit hyperemia, edema, and hemorrhages (9, 10). In pregnant ewes, SPPV infection can result in abortion or the birth of nonviable offspring. The course of the disease typically lasts 20–30 days, but it may be prolonged in immunocompromised animals. Mortality reaches 50–80% in lambs and generally ranges from 5 to 10% in adult sheep (4, 8, 10).

Pathological examinations often reveal less pronounced skin lesions than those observed clinically. Necrosis of mucosal surfaces and generalized lymphadenopathy are typical. Papules and ulcers are frequently detected on the abomasal mucosa and occasionally on the rumen, large intestine, tongue, palate, and esophagus. Pale foci up to 2 cm in diameter may be observed on the liver and kidneys. The lungs commonly show multiple firm nodular formations up to 5 cm in diameter, especially near the diaphragm (11, 12).

Epidemiological data from the World Organization for Animal Health (WOAH) indicate reports of sheeppox outbreaks in several countries in 2021, including Turkey, Israel, China, Mongolia, Thailand, Russia, Algeria, Kenya, Tunisia, and Uganda (13). More recently, in 2023–2024, new cases were documented in regions of Russia (Vladimir, Moscow, Astrakhan, Oryol, and Tyumen Oblast) and Greece (Macedonia and Thrace) (14, 15). In Kazakhstan, outbreaks were recorded in 2019 in the rural area of Kyzylözen in Tupkaragan District in the Mangystau Region, as well as the village of Suyunduk in Kurmangazy District in the Atyrau Region. Prompt action by veterinary authorities successfully contained these outbreaks (16).

Since 2019, no officially confirmed cases of sheeppox have been reported in Kazakhstan. In 2024, clinical signs suggestive of sheeppox were observed in individual sheep from a flock in the Almaty Region, which has prompted further investigation. Laboratory investigations led to the isolation of a virus from pathological material, which was morphologically and molecularly identified as SPPV (unpublished data). This finding suggests the possible persistence of the virus within the local sheep population and highlights its potential epizootic risk.

In light of these developments, detailed investigation of the isolated virus is essential to assess its genetic characteristics and virulence. Phylogenetic analysis was conducted to determine the origin of the strain, establish its relatedness to known isolates from different countries, and compare its pathogenicity with that of well-characterized SPPV strains. The data obtained could help to improve the understanding of the current epizootic situation in the region, support risk assessments for further spread, and aid in the development of effective prevention and control strategies.

## 2 Methods

### 2.1 Virus

This study examined the SPPV isolate “Sheeppox/KZ/Targap/2024,” which was obtained in 2024 from a clinically affected lamb in the village of Targap in the Zhambyl District of the Almaty Region. Some of the animals in this village showed clinical signs suggestive of sheeppox that warranted further laboratory investigation. The isolate was subjected to three serial passages in lamb kidney cell (LKC) culture. A 50% tissue culture infectious dose (TCID_50_) assay indicated a viral titer of 5.33 ± 0.08 log_10_ TCID_50_/mL. The virus was identified using polymerase chain reaction (PCR) and next-generation sequencing (NGS). The molecular and genetic characteristics of the complete genome of the “Sheeppox/KZ/Targap/2024” strain have been deposited in the GenBank database (accession ID: PV434148).

### 2.2 Isolation and titration of the virus in cell culture

For the isolation of SPPV, clinical specimens were collected from diseased animals in the form of a 20% homogenate of affected skin tissue. To reduce bacterial contamination, the homogenate was pretreated with a combination of antibiotics (benzylpenicillin at a concentration of 1,000 U/mL and streptomycin at 100 μg/mL). Subsequently, 200 μL of the prepared 20% virus-containing homogenate were inoculated into culture flasks containing LKCs according to a previously described protocol (17). After inoculation, the cells were incubated at 37°C for 1 h to facilitate viral adsorption. The inoculum was then removed and replaced with fresh maintenance medium. The cultures were incubated further at 37°C for 5–7 days with daily microscopic observations for cytopathic effect (CPE) to ensure optimal conditions for virus replication.

Virus titration was performed in 96-well plates using LKC monolayers and 10-fold serial dilutions of the viral suspension in accordance with established methods (17, 18). The plates were incubated at 37°C for 7 days with daily examination for CPE. CPE was assessed using an inverted microscope, and morphological alterations such as cell rounding, vacuolization, and lysis were recorded. The supernatants from cultures exhibiting CPE in more than 80% of cells were harvested and used for subsequent passages. Viral titers (TCID_50_/mL) were determined using the Reed and Muench method (19), in which wells exhibiting one or more viral plaques were considered positive.

### 2.3 Animals and bioethics

The pathogenicity of the SPPV isolate was evaluated in 10 clinically healthy Merino sheep aged 6–12 months with body weight of 17–20 kg. The animals were sourced from farms that were certified as being free of acute infectious diseases and were seronegative for SPPV, which was confirmed by preliminary serological testing. Prior to the experimental procedures, all animals were individually identified and subjected to a 30-day quarantine period for observation and acclimatization.

All experimental procedures were conducted in a biosafety level 3 animal facility (ABSL-3), which was equipped with high-efficiency particulate air (HEPA) filtration systems for both incoming and outgoing air, a pass-through autoclave for decontamination, and a local sanitary checkpoint with a mandatory shower for personnel. Animal housing, care, and feeding were performed in strict accordance with established guidelines and regulatory instructions (20, 21). All procedures involving animals were conducted in accordance with the Law of the Republic of Kazakhstan On Responsible Treatment of Animals (Law No. 97-VII ZRK, December 30, 2021), as well as other applicable national and institutional guidelines. The experimental protocol was reviewed and approved by the Animal Experimentation Ethics Committee of LLP Research Institute for Biological Safety Problems (Approval No. 1, dated July 14, 2024).

### 2.4 Study design

The experimental animals were randomly allocated to two groups. The first group (*n =* 5) was intradermally inoculated with the SPPV isolate “Sheeppox/KZ/Targap/2024.” The inoculation was performed on both the left and right sides of the body with each viral dilutions of 10^−1^ to 10^−7^ administered at four distinct sites with a volume of 250 μL per site. The second group (*n =* 5) served as an uninfected control.

All animals were clinically monitored daily for a period of 14 days, which included measurements of rectal body temperature. A sterile vacuum-based sampling system was used according to collect nasal swabs, whole blood (for virological and molecular genetic analyses), and blood serum (for ELISA and virus neutralization testing) on days 3, 5, 7, 9, 11, and 14 post-inoculation. On day 14, pock lesions were collected from the skin of clinically affected animals along with prescapular and submandibular lymph nodes for virological examination. The specificity of the disease was determined based on characteristic clinical signs, virus isolation, genome detection, and the presence of virus-neutralizing antibodies in serum. The course and development of the disease were assessed by evaluating the nature, diversity, and severity of clinical manifestations.

### 2.5 Serum neutralization test (SNT)

To detect SPPV-neutralizing antibody titers, an SNT was performed as described in the OIE Terrestrial Manual for Diagnostic Tests and Vaccines (OIE, 2012). Virus neutralizing antibody (VNA) titers were estimated against a value of 100 TCID_50_ for the SPPV isolate (Sheeppox/KZ/Targap/2024). SPPV-specific positive and negative antisera controls were included. Briefly, sera were diluted (1:2 to 1:128) in 96-well flat-bottom microtiter plates, and an amount equivalent to 100 TCID_50_ of the SPPV field isolate was added to the wells. Plates were incubated for 1 h at 37°C and kept overnight at 4°C.

After incubation, 50 μL of LKC suspension containing 2 × 10^5^ cells/mL were added to each well, and the plates were incubated for 4–7 days at 37°C in a 5% CO_2_ atmosphere. The plates were then screened for the presence of SPPV-induced CPE. Neutralization titers were determined as the inverse of sera dilution, giving the 50% neutralization end-point.

### 2.6 Enzyme-linked immunosorbent assay (ELISA)

Specific antibodies against SPPV were detected using a commercial ELISA kit (ID.vet Screen® Capripox Double Antigen Multi-species, France) in accordance with the manufacturer’s instructions. Optical density (OD) was measured at a wavelength of 450 nm using a Chromate®-4300 microplate reader (Awareness Technology, USA). Serum samples with an OD value less than 30% of the mean value of the negative control (S/N) were interpreted as negative, while samples with OD values greater than 30% were considered positive for antibodies against SPPV.

### 2.7 DNA extraction

Genomic DNA was extracted using the DNeasy® Blood & Tissue Kit (250) (QIAGEN, Germany) in accordance with the manufacturer’s instructions.

### 2.8 Detection of Capripoxvirus DNA by real-time PCR

Detection of sheeppox virus DNA was carried out using the commercial ID Gene™ Capripox Triplex kit (ID Vet, France) following the manufacturer’s instructions.

Each 25 μL reaction contained 15 μL of Master Mix, 5 μL of extracted DNA, and 5 μL of nuclease-free water. Amplification was performed on the Rotor-Gene Q thermocycler (Qiagen, Germany) using the following cycling conditions: 95°C for 10 min, followed by 45 cycles of 95°C for 15 s and 60°C for 30 s.

Fluorescence was detected in the FAM, HEX/VIC, and Cy5 channels. Results were interpreted in accordance with the ID Vet guidelines. A sample was considered positive if a characteristic amplification curve with a Ct value in the valid range was obtained.

### 2.9 Library preparation and whole-genome sequencing

Viral DNA was extracted from virus-containing fluid using the innuPREP Virus DNA/RNA Kit (Analytik Jena AG, Germany) according to the manufacturer’s protocol. The DNA concentration was measured using the Qubit DNA HS Assay Kit (Life Technologies, Carlsbad, CA, USA) on a Qubit 2.0 fluorometer (Life Technologies, Carlsbad, CA, USA) in accordance with the manufacturer’s instructions. For library preparation, 20 μL of viral DNA at a concentration of 50 ng/μL were used. DNA libraries were generated through enzymatic fragmentation and ligation using the Ion Plus Fragment Library Kit (Thermo Fisher Scientific, USA). Purification steps were performed using Agencourt AMPure XP magnetic beads (Beckman Coulter, USA) according to the manufacturers’ protocols.

Fragmented DNA was separated according to size using horizontal electrophoresis on 1.5% agarose gel (Sigma, USA) stained with ethidium bromide. The gel was run in tris-acetate buffer at a voltage of 100 V/cm for 30 min. DNA fragments ranging from 350 to 500 bp were excised under UV visualization using an Invitrogen iBright CL1500 transilluminator (Thermo Fisher Scientific, Inc., Rockford, USA). 1-kb DNA Ladder ready-to-use (Bioron, Germany) was used as the molecular weight marker.

Gel-purified DNA was recovered using the innuPREP DOUBLEpure Kit (Analytik Jena AG, Germany). Library amplification was performed using components from the Ion Plus Fragment Library Kit according to the manufacturer’s instructions. Amplified libraries were quantified using the Ion Universal Library Quantitation Kit. Template preparation and chip loading were performed using the Ion Chef System with the Ion 510, Ion 520, and Ion 530 Kits and an Ion 530 chip (Thermo Fisher Scientific). Whole-genome sequencing of the virus was conducted using the Ion Torrent platform on the Ion GeneStudio S5 sequencer. Sequencing results were generated using Ion Torrent Suite Software version 5.12 and exported in UBAM and FASTQ file formats.

### 2.10 Genome assembly

Genome assembly was performed using the UGENE software suite version 52. The input data consisted of NGS reads in the FASTQ format. A reference-guided assembly approach was employed using the genome sequence of Sheeppox virus 17077-99 (NCBI Reference Sequence: NC_004002.1) as a reference. The alignment and assembly procedures used the default parameters recommended by the software.

### 2.11 Phylogenetic analysis

For the phylogenetic analysis, the complete genome sequence of the studied isolate Sheeppox/KZ/Target/2024 was aligned with genome-wide sequences of other Capripoxvirus representatives retrieved from the NCBI database. The evolutionary history was inferred using the Maximum Likelihood method based on the Tamura–Nei model (22) and the analysis was performed in MEGA11 (23). To assess the reliability of the resulting tree topology, 1,000 bootstrap replicates were conducted. Although the use of an outgroup was initially considered, it was excluded from the final tree construction because its inclusion distorted the topology and reduced bootstrap support for internal nodes. Therefore, to improve the clarity of the phylogenetic relationships among the target sequences, the final tree was generated without an outgroup.

### 2.12 Histopathological examination

Tissue samples were collected and documented from the affected skin lesions (pocks), lymph nodes, spleen, lungs, and rumen. Samples were obtained from different animals using sterile surgical scalpels and artery forceps by excisional biopsy (2–4 g). They were then immediately placed into sterile disposable containers containing 10% neutral buffered formalin for fixation and subsequent histopathological processing in accordance with established protocols (24, 25). The tissues were subjected to standard histological processing. Paraffin-embedded tissue blocks were sectioned at a thickness of 5 μm, and for general histopathological evaluation, the sections were stained with hematoxylin and eosin (H&E).

### 2.13 Statistical analysis

All statistical analyses were performed using GraphPad Prism® version 9.0 (GraphPad Software, Inc., La Jolla, CA, USA). Pairwise correlation analysis was used to evaluate the relationship between clinical disease manifestations and the development of humoral immunity in the post-challenge groups. Arithmetic means and standard errors of the measured parameters were calculated. A two-way analysis of variance (ANOVA) was applied to compare rectal temperatures, clinical scores, and serological responses between the experimental and control groups. A *p*-value of ≤ 0.05 was considered statistically significant.

## 3 Results

### 3.1 Isolation of SPPV in cell culture

**Figure 1:**
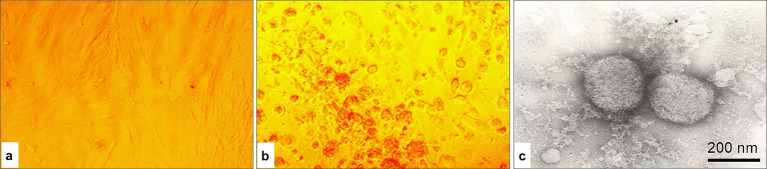
Cytopathic effect in LKC culture and electron microscope images of the SPPV isolate: **(a)** uninfected control LKC culture; **(b)** LKC culture infected with SPPV; **(c)** negatively stained viral particles using 2% phosphotungstic acid (PTA), 90,000× magnification.

Following infection of the LKC monolayer with a 20% homogenate of affected skin tissue, the initial CPE of the SPPV was observed at 48–72 h post-infection. The changes were characterized by the appearance of shiny, light-refracting, rounded cells elevated above the monolayer surface. By 144–168 h post-infection, the extent of monolayer disruption reached approximately 80–90% (Figure 1). Electron microscope analysis of the SPPV revealed the presence of spherical virions with diameters ranging from 240 to 280 nm in the examined material. When the infectious activity of the isolated SPPV was assessed in LKC culture, the average viral titer was determined to be 5.33 ± 0.08 log_10_ TCID_50_/mL.

### 3.2 Phylogenetic analysis

**Figure 2:**
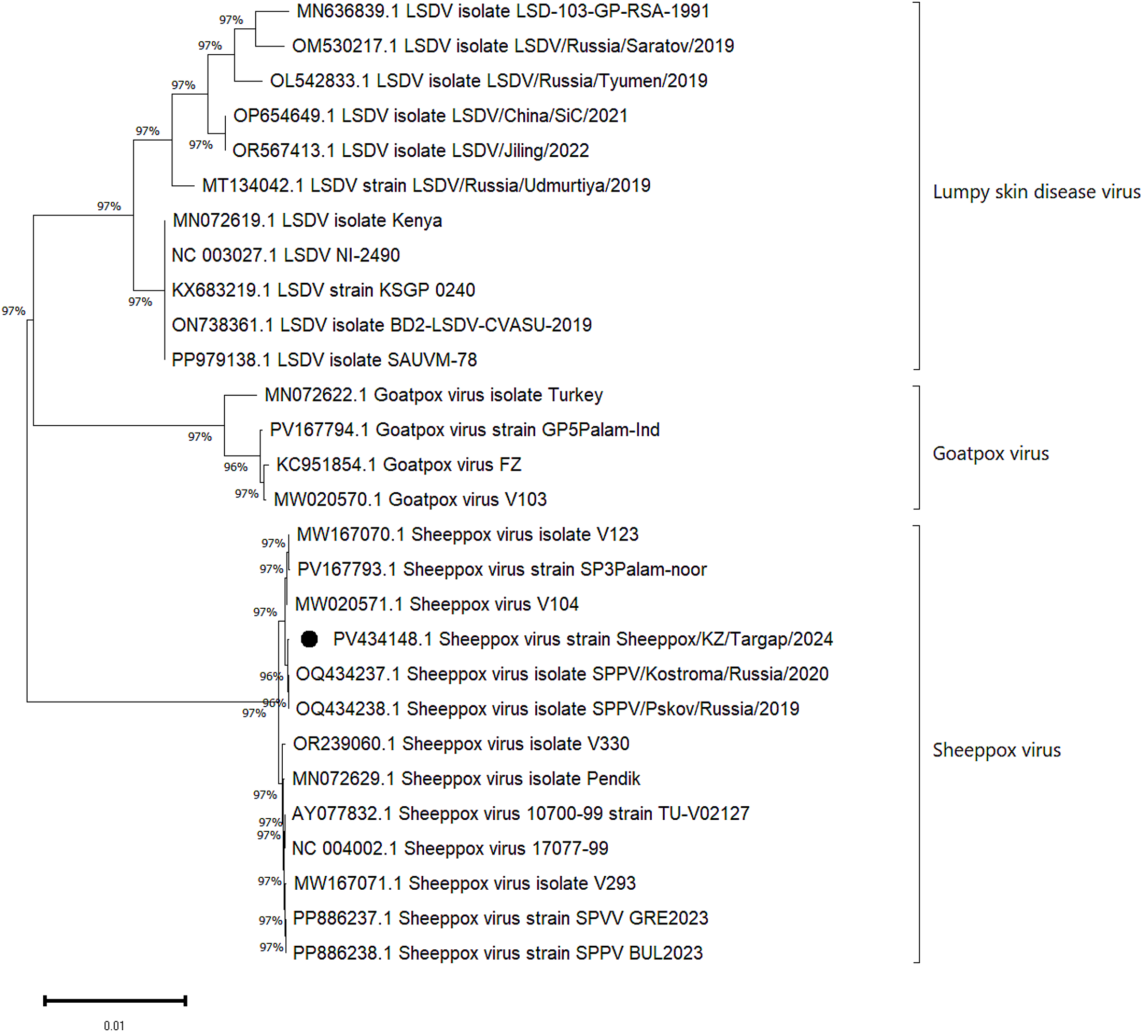
Phylogenetic analysis of the Sheeppox/KZ/Targap/2024 isolate (indicated by a black circle) alongside 27 global strains from various members of the family Poxviridae, subfamily Chordopoxvirinae, and genus *Capripoxvirus* retrieved from the NCBI GenBank database. The x-axis represents the tree scale.

Based on the phylogenetic analysis (Figure 2), it was established that the Sheeppox/KZ/Targap/2024 isolate belongs to the SPPV species, which is part of the subfamily Chordopoxvirinae. The formed a monophyletic group (bootstrap (BS) = 96% and (BS) = 97%) with samples OQ434237.1 (SPPV isolate SPPV/Kostroma/Russia/2020), OQ434238.1 (SPPV isolate SPPV/Pskov/Russia/2019) and MW167070.1 (SPPV isolate V123), MW1020571.1 (SPPV isolate V104), and PV167793.1 (SPPV strain SP3Palam-noor). Analysis using the BLASTN program (NCBI) showed that the examined isolate also had a high degree of similarity (99.94%) with an Indian isolate (PV167793.1) and Russian isolates (OQ434238.1 and OQ434237.1).

### 3.3 Clinical signs and pathogenicity of the virus

**Figure 3:**
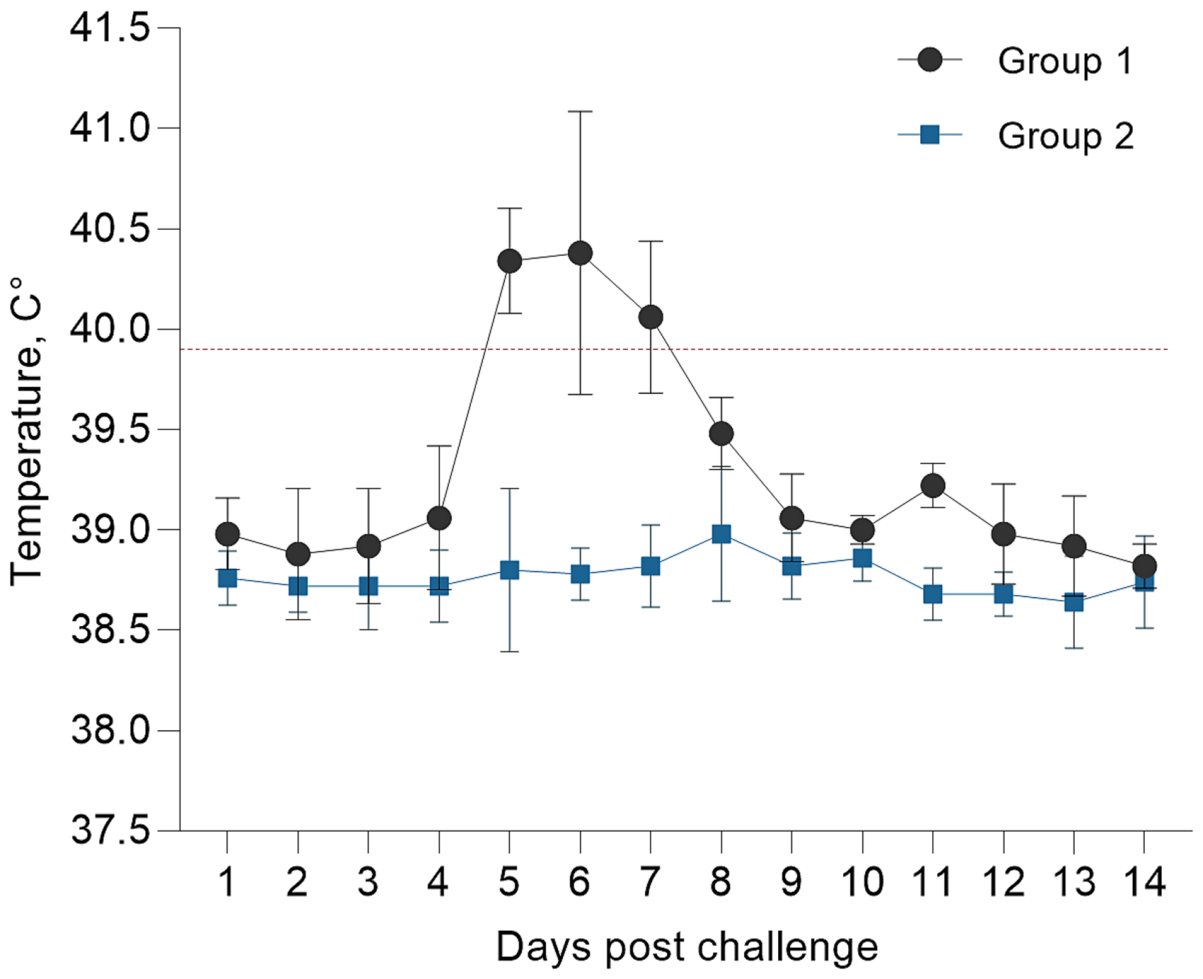
Dynamics of body temperature in infected animals.

The rectal temperatures recorded daily for 14 days post-infection are presented as the mean ± standard error for the infected group (*n =* 5). Body temperatures showed a marked increase on days 5–6 post-infection and peaked at 41.4–41.8°C, with hyperthermia persisting for approximately 3 days (Figure 3). The control group (*n =* 5) showed no significant changes in temperature throughout the observation period.

**Figure 4:**
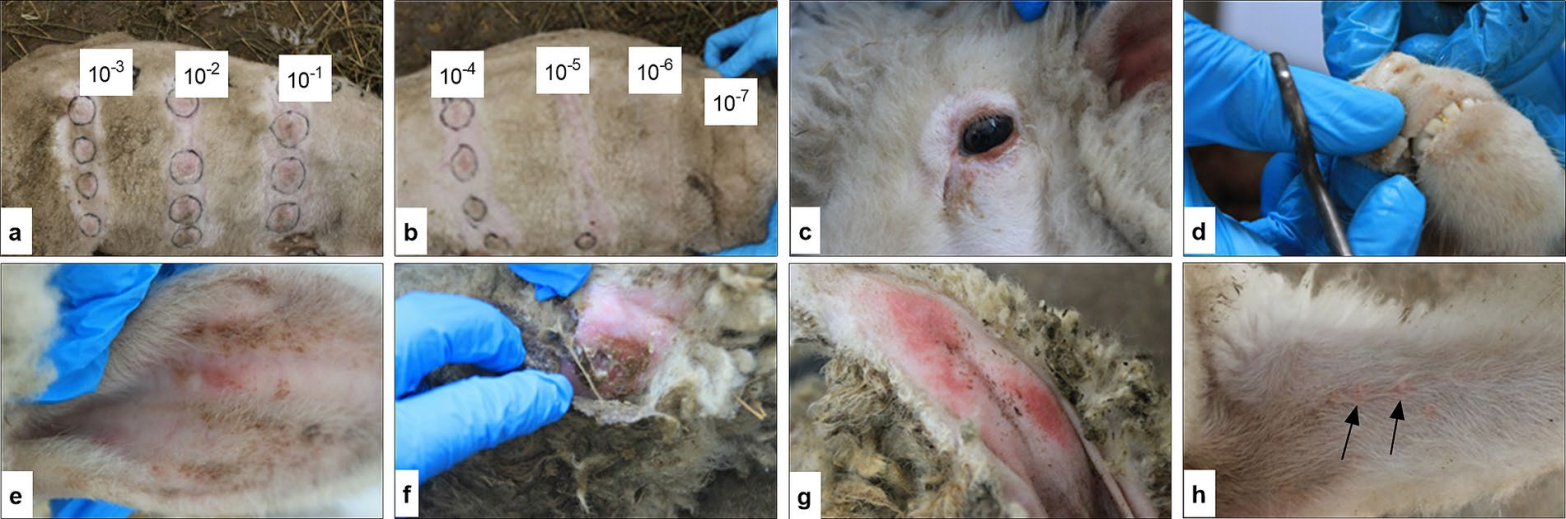
Clinical response to SPPV inoculation during pathogenicity assessment. Inoculation sites on the right and left sides of the sheep’s body with viral dilutions from 10^−1^ to 10^−7^ **(a,b)**. Roseolar and papular stages of lesions on the head and around the eyes **(c,d)**. Exanthema in the roseolar stage in the ear region **(e)**. Papular stage with signs of suppuration on the skin **(f)**. Roseolar-papular lesions on sparsely haired areas of the tail **(g)**. Generalized papular eruptions on the forelimbs and hindlimbs **(h)**.

Erythema developed on days 7–9 post-infection along with catarrhal inflammation of the mucous membranes and papular-pustular skin lesions. The papules appeared as firm, tumor-like nodules measuring 0.5–1.0 cm in diameter with distribution across the entire body surface, including the inner surfaces of the forelimbs and hindlimbs (Figure 4h). Characteristic pox lesions also occurred on the head, lips, and around the eyes (Figures 4a–g).

Infected sheep exhibited signs of systemic intoxication beginning on days 3–5 post-infection, including lethargy and reduced appetite, which are characteristic of the clinical course of sheeppox. The papular stage persisted for approximately 5–7 days. The sites of viral inoculation showed localized skin induration and erythema with lesions reaching up to 4 cm in diameter. The activity of the examined isolate at the inoculation sites was determined to be 4.75 ± 0.02 log_10_ ID_50_/cm^3^ (Figures 4a,b).

### 3.4 Serum neutralization test

**Figure 5:**
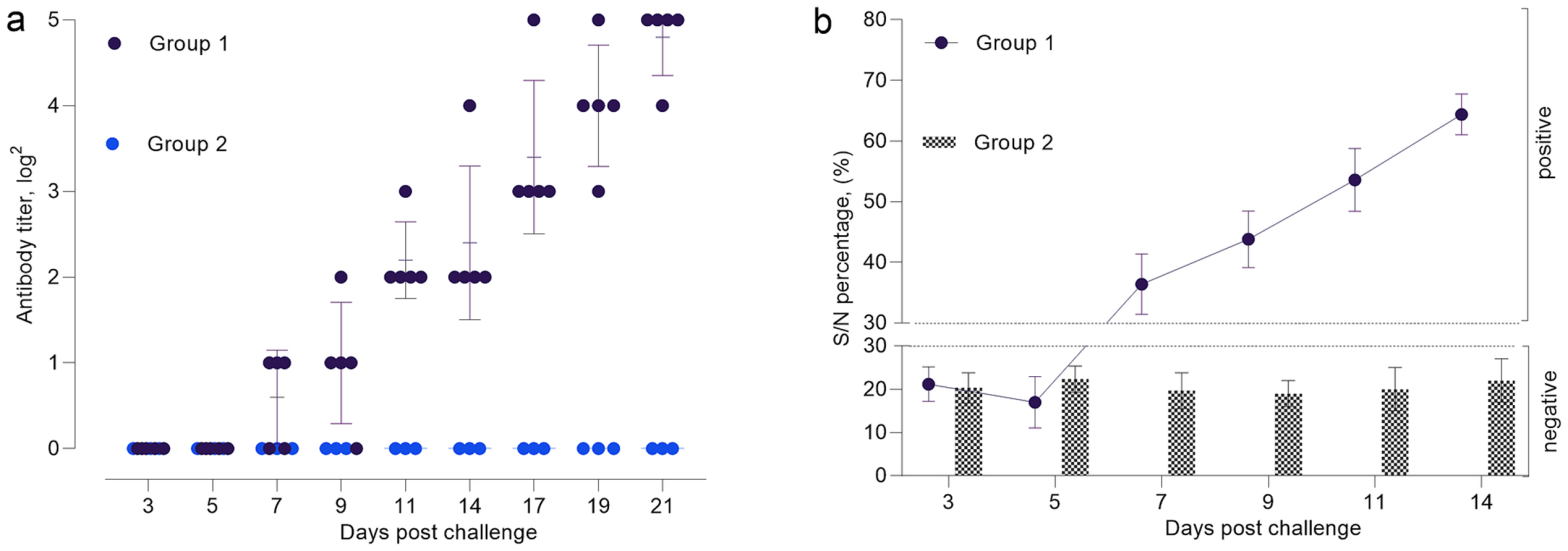
Antibody response dynamics in experimental animals following infection with SPPV: **(a)** neutralizing antibody titers determined by virus neutralization test (VNT); **(b)** percentage inhibition of specific antibodies measured by ELISA in serum samples.

The VNA titers of were measured in the experimental groups to assess the dynamics of the humoral immune response (Figure 5a). According to the virus neutralization test (VNT), neutralizing antibodies were detected in 60% of the sheep by day 7 post-infection. By day 9, seroconversion was observed in all animals (100%), and by day 21, antibody titers reached their peak levels (1:32), indicating a robust immune response.

### 3.5 Elisa

On days 3 and 5 post-infection, antibody levels in all animals remained below the threshold value of 30%, which indicated the absence of a significant immune response. By day 7, antibody titers in several animals reached approximately 40%, which suggested the onset of seroconversion. On days 9 and 11, most animals exhibited antibody levels exceeding the threshold (S/*p* ≥ 30%), with values reaching 70% or higher in a substantial proportion of the group. These results indicate activation of the immune system and the production of specific antibodies against the Sheeppox virus. Starting from day 14 post-infection, antibody levels continued to increase steadily and reached peak values by day 21. At this stage, all animals exhibited pronounced seroconversion with antibody levels exceeding 90%, indicating a strong and robust immune response (Figure 5b).

### 3.6 PCR and pathological examination

**Figure 6:**
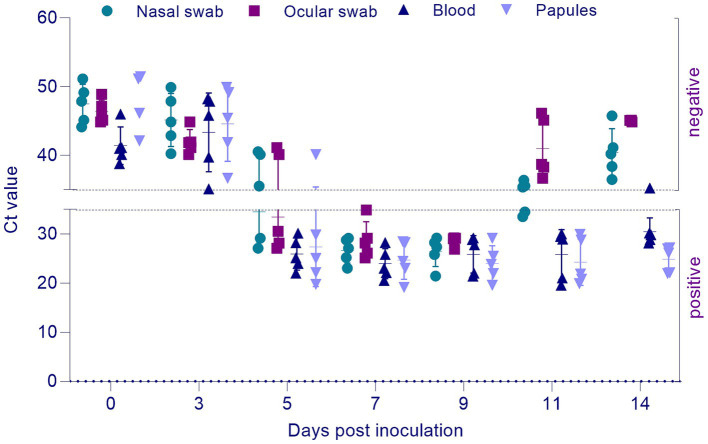
PCR detection of SPPV DNA. Ct values ≤35 were interpreted as positive, while Ct values >35 were considered negative.

**Figure 7:**
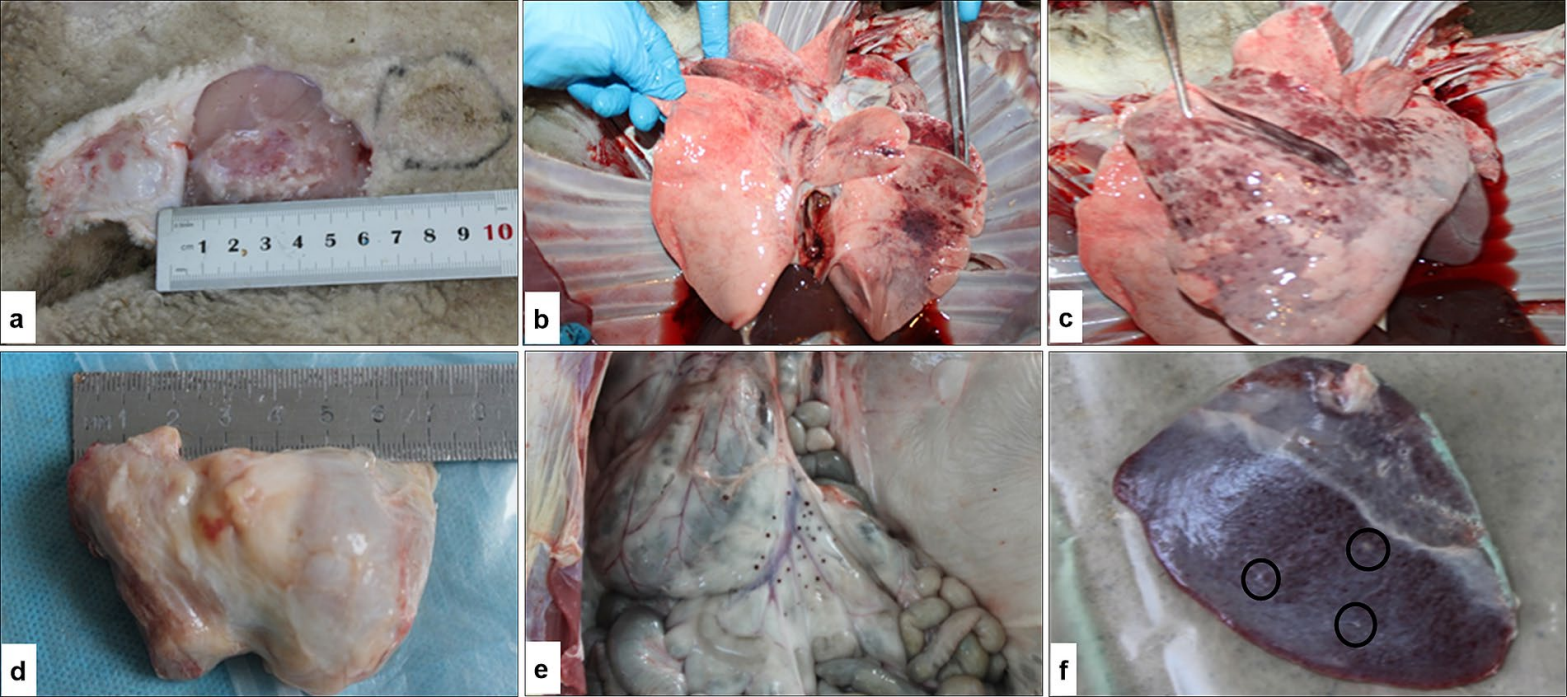
Gross pathological findings in sheep following experimental infection with SPPV: **(a)** inoculation sites on the right and left sides of the body with viral dilutions from 10^−1^ to 10^−7^; **(b,c)** focal lesions in the lungs characteristic of sheeppox infection; **(d)** enlarged, congested, and hemorrhagic prescapular lymph node; **(e)** multiple nodular lesions on the mucosal surface of the small intestine; **(f)** round, grayish-white plaques on the surface of the spleen.

Viral DNA of the SPPV was first detected on day 5 post-infection in nearly all tissues of the infected sheep. By days 11 and 14, viral DNA was no longer detectable in nasal and ocular swabs. Nevertheless, the viral DNA remained consistently present in whole blood and affected skin tissue samples throughout all time points of the study (see Figure 6). Post-mortem examination conducted on day 14 post-infection revealed pathological changes in the internal organs, including the lungs, spleen, submandibular lymph nodes, and skin, as shown in Figure 7. The changes were consistent with the characteristic lesions of sheeppox. Typical findings included the presence of pox lesions (pocks), tissue edema, congestion, and vascular thrombosis across the examined organ systems.

### 3.7 Histopathological changes in the skin and other organs

**Figure 8:**
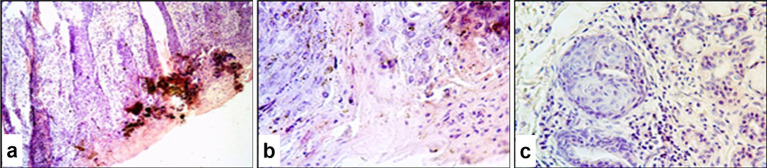
Paraffin-embedded section of sheep skin affected by SPPV. **(a)** General microscopic view of the skin showing epidermal and dermal necrosis, hyperkeratosis of epidermal glands, and hair follicle involvement; **(b)** epithelial alteration and epidermal hyperkeratosis; **(c)** structural damage to dermal skin glands. Hematoxylin and eosin staining, 400× magnification.

Microscopically, the affected skin areas were readily distinguishable (Figures 8a,b). In these regions, the epidermis had dissolved and formed a yellow to dark-red amorphous mass. Intercellular adhesion between keratinocytes was lost, and detached epithelial cells were observed floating in serous exudate. A homogeneous necrotic zone extended into the mid-dermal layers.

Many skin regions showed marked surface hyperkeratosis (excessive thickening of the stratum corneum). This was accompanied by pronounced proliferative activity of the keratinized epithelial cells, which led to significant epidermal thickening ranging from several millimeters to several centimeters in some areas. Intracellular fluid accumulation occurred in the basal and spinous layers of the epidermis, which resulted in the formation of microvesicles of various sizes filled with a homogeneous pink-stained matrix and characteristic poxvirus-infected cells.

The glandular epithelium of the skin also exhibited structural changes, including thickening of the follicular walls up to several cell layers (Figure 8c). Similar pathological alterations occurred in the architecture of hair follicles. Inflammatory cells were present both in clusters and diffusely surrounding the affected tissue. Along with other structural skin cells, many of these exhibited signs of apoptosis with numerous apoptotic bodies.

### 3.8 Histopathological changes in the lungs

**Figure 9:**
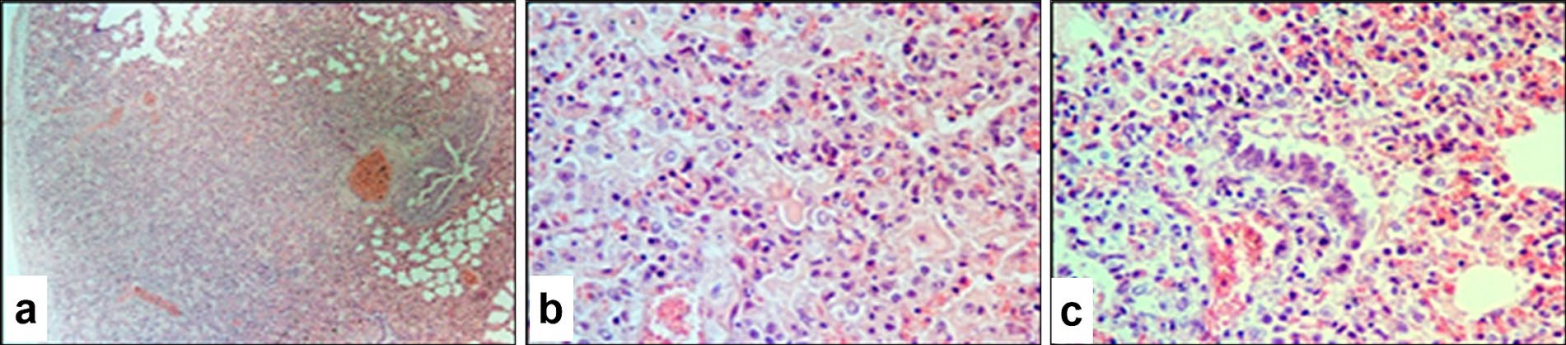
Paraffin-embedded section of lung tissue affected by SPPV. **(a)** General view of a pox (atypical) nodule in the lung at low magnification; **(b)** vacuolated cells (Bollinger bodies) with and without characteristic viral inclusion bodies, hemorrhage into the pulmonary parenchyma, and individual apoptotic cells with visible apoptotic bodies; **(c)** atypical changes in the bronchiolar epithelium and extensive hemorrhage in the lung parenchyma. Hematoxylin and eosin staining, 400× magnification.

Microscopic examination of the lungs of infected sheep revealed characteristic pox nodules located beneath the pleural surface (Figure 9a). These nodules were clearly demarcated from the surrounding healthy tissue and were characterized by alveolar loss, pleural thickening, and marked inflammatory changes. The pox nodules were predominantly composed of vacuolated pox-infected cells, modified fibroblasts, alveolar macrophages, lymphocytes, and neutrophils. Viral inclusion bodies were observed in both the nuclei and cytoplasm of pox-infected cells and fibroblasts (Figure 9b). A significant proportion of cells showed features of apoptosis evidenced by the presence of numerous apoptotic bodies.

Notable alterations were also found in the epithelium of small bronchi and bronchioles (Figure 9c), including epithelial hyperplasia, stratification, nuclear swelling, and the presence of viral inclusions. The connective tissue stroma of the pleura was loosened and contained numerous swollen fibroblasts, many of which harbored eosinophilic viral inclusion bodies. In certain areas, fibroblasts formed dense fibrous structures with signs of tissue invasion.

### 3.9 Histopathological changes in the rumen

**Figure 10:**
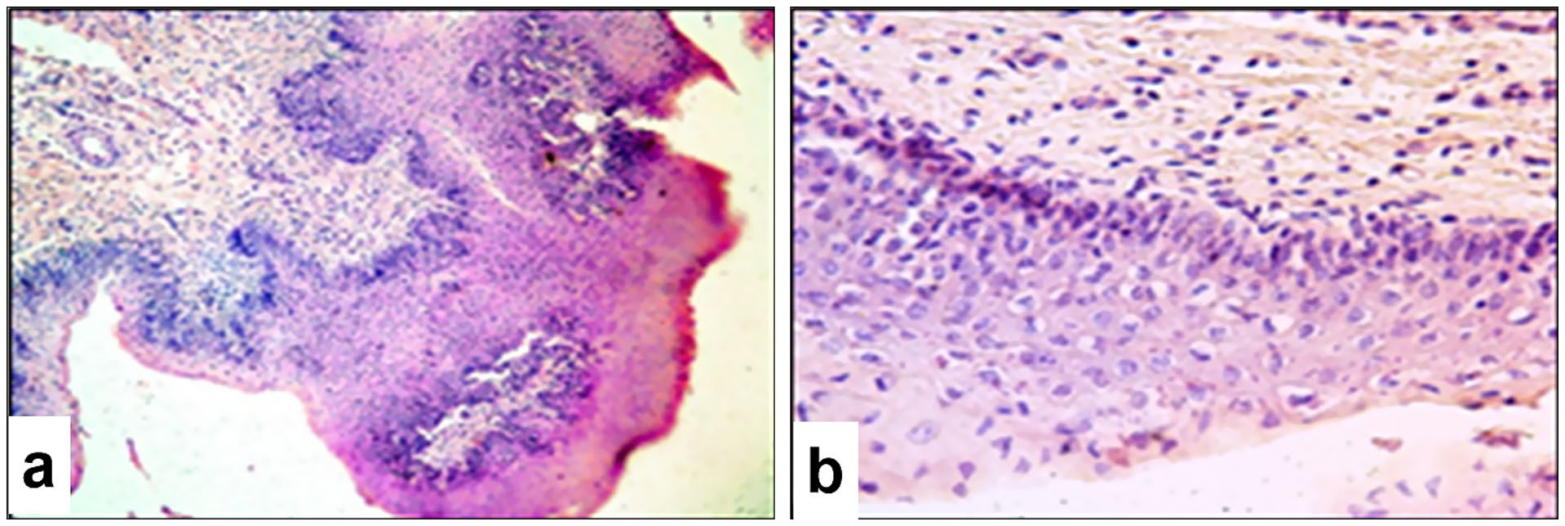
Paraffin-embedded section of the rumen affected by SPPV. **(a)** Polyp-like protrusion and hyperkeratosis of the ruminal mucosal epithelium with numerous pox-infected cells; **(b)** multiple pox-infected cells in the surface epithelium of the rumen. Hematoxylin and eosin staining, 400× magnification.

Microscopic examination of the pathological protrusions in the rumen revealed hyperplasia of the stratified non-keratinizing epithelium, which was accompanied by pronounced proliferative activity in the basal layer evidenced by intense cellular staining and the presence of mitotic figures (Figure 10a). In some areas, basal layer disruption was observed with the formation of multiple layers of epithelial cells. The submucosa was thickened and contained swollen fibers, modified fibroblasts, and diffuse infiltration by inflammatory cells. Cells of the spinous layer were increased in number with vacuolated nuclei, prominent nucleoli, and diffuse chromatin patterns. Intracytoplasmic eosinophilic inclusion bodies were observed in some cells (Figure 10b). The superficial layer showed signs of hyperkeratosis and partial destruction of the keratinized material, and individual cells exhibited features of apoptosis.

### 3.10 Histopathological changes in the lymph nodes

**Figure 11:**
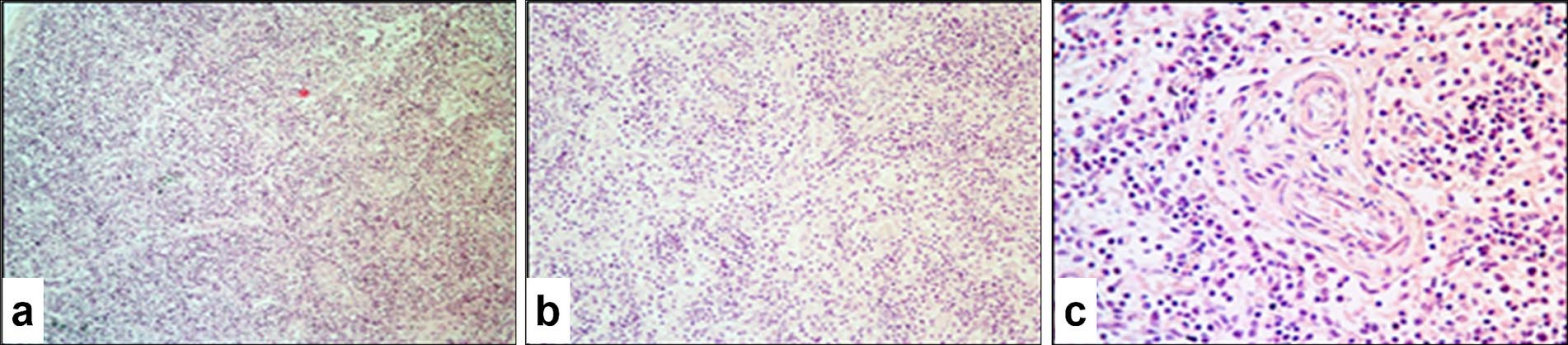
Paraffin-embedded section of a lymph node affected by SPPV. **(a)** Marked depletion of lymphoid follicles and the paracortical zone of the cortex; **(b)** medullary region sparsely populated with lymphocytes, large epithelial-like cells, macrophages, and plasma cells; **(c)** destructive changes in blood vessels and accumulation of immune cells surrounding them. Hematoxylin and eosin staining, 200× magnification.

Infection of the lymph nodes with SPPV resulted in pronounced structural alterations characterized predominantly by destructive and inflammatory processes. Lymphoid elements were diffusely distributed with no clear demarcation between the cortical and medullary regions (Figure 11a). The organ capsule contained swollen fibroblasts with eosinophilic viral inclusion bodies, particularly near the follicular zone. The follicular (B-cell) zone appeared depleted with lymphoid follicles replaced by epithelial-like cells, fibroblasts, and macrophages, and many of them contained intracytoplasmic eosinophilic viral inclusions. The number of lymphocytes and plasma cells was reduced, and a proportion of these cells showed morphological features of apoptosis.

The paracortical zone displayed scattered clusters of immunocompetent cells, which mainly surrounded infected cells. Mitotic activity was low, while apoptosis was prominent, as evidenced by the presence of apoptotic bodies. In the medullary region, cords composed of lymphocytes, macrophages, and epithelial-like cells were preserved. Fibroblast infection was associated with alterations in vascular walls, which indicated the systemic nature of viral invasion (Figures 11b,c).

### 3.11 Histopathological changes in the spleen

**Figure 12:**
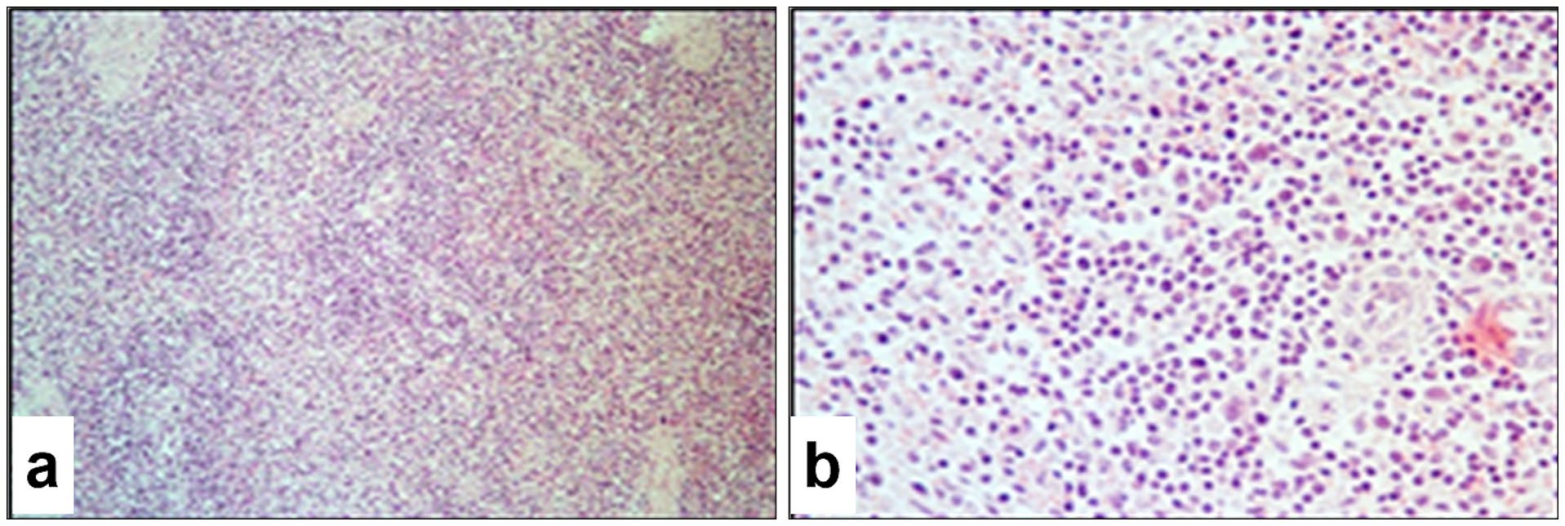
Paraffin-embedded sections of spleen tissue affected by SPPV. **(a)** T-cell zone of the white pulp loosely populated with diffuse lymphoid tissue and various cellular elements; the B-cell zone appears atrophic. **(b)** Cells of the red pulp, the majority of which contain intracytoplasmic or intranuclear eosinophilic viral inclusion bodies. Hematoxylin and eosin staining, 400× magnification.

The histopathological appearance of the spleen in sheep infected with SPPV closely resembled that of the lymph nodes and showed pronounced destructive processes affecting the stromal architecture. Swollen fibroblasts containing eosinophilic viral inclusion bodies were observed in the capsule, trabeculae, and vascular walls, which predominantly occurred within the loose connective tissue of the red pulp. The boundaries between red and white pulp were indistinct. In the white pulp, lymphoid follicles appeared atrophic with reduced lymphocyte counts, and isolated virus-infected cells were detected (Figure 12a). The T-cell zones were relatively preserved, although cellular proliferative activity was minimal, and a high number of apoptotic lymphocytes was evident. Throughout the pulp, epithelial-like cells and fibroblasts containing viral inclusion bodies predominated (Figure 12b), and macrophages and plasma cells were diffusely distributed but sparse.

## 4 Discussion

The present study focuses on the molecular-genetic and pathogenetic characterization of an SPPV isolate that was obtained from clinically affected animals in the Republic of Kazakhstan. The data obtained are of considerable importance for evaluating the effectiveness of national preventive measures that are currently being implemented. Despite routine vaccination campaigns, cases of sheeppox continue to be reported, including cases among Merino sheep, the main productive breed that is widely distributed in the southern regions of Kazakhstan (16, 26). These occurrences may be attributed to failures at various stages of the vaccination process, such as improper transport or storage conditions, or an absence of vaccination in certain farms. Nevertheless, the vaccine currently in use (Niskhi strain) has demonstrated long-term efficacy against sheeppox for over 30 years (27, 28) and has also shown cross-protective effectiveness against lumpy skin disease virus in cattle (29).

We isolated the virus and studied its biological properties using LKC culture, which has proven to be a reliable model for propagating SPPV. This system also enables assessment of the interactions between the virus and host cells and the virus-induced CPE. The observed CPE characteristics are consistent with previously published findings (30), which confirms the reproducibility of the model. For instance, one study (31) reported a viral titer of 5.33 ± 0.08 log_10_ TCID_50_/mL in LKCs at the third passage, whereas 4.75 ± 0.02 log_10_ ID_50_/mL was obtained with sheep skin tissue, which indicates that LKCs have higher sensitivity to SPPV.

The isolated virus was successfully adapted and propagated in the LKC culture system, which confirmed its suitability for laboratory cultivation and further virological studies. Electron microscope examination of the viral particles revealed virions with a rounded or brick-shaped morphology, which is consistent with previously described ultrastructural characteristics (32). Phylogenetic analysis of the investigated isolate demonstrated a high degree of nucleotide identity (99.94%) with isolates identified in the Russian Federation (33, 34). This finding may indicate a common origin or a close epizootiological relationship among SPPV strains circulating within the region. The results also confirm the classification of the Kazakhstani isolate as a member of the SPPV species, which is consistent with the morphological features observed via electron microscopy (33, 34).

To confirm a diagnosis of sheeppox, clinical material should be collected from characteristic lesions, including fresh papules on the skin prior to suppuration, affected lung tissue, the spleen, lymph nodes, and blood samples (35). The virus exhibits systemic dissemination within the host and primarily targets organs involved in the formation and transport of physiological fluids such as blood, saliva, and semen. Sheep and goats of all ages are susceptible to SPPV infection, although the disease tends to be particularly severe in young animals (36). It is important to note that the pathogenicity of the virus may vary according to the specific isolate and its geographic origin. This highlights the necessity of evaluating the clinical manifestations and virulence of regional strains, including the Kazakhstani isolate characterized in this study.

According to Hajjou and Khataby (30), clinical signs of sheeppox in sheep typically appear between days 3 and 5 post-infection and peak between days 14 and 21. The isolate used in the present study induced observable clinical symptoms as early as day 5 post-infection, indicating high virulence and pathogenic aggressiveness. Experimental infection of sheep with the isolated strain was accompanied by pronounced clinical signs indicating high pathogenicity. The animals exhibited typical symptoms including elevated body temperature, lethargy, anorexia, and generalized skin lesions. These findings suggest that the isolate could be capable of inducing a severe form of the disease in sheep, including generalized manifestations, particularly in susceptible breeds.

Immunological studies demonstrated that specific VNAs began to develop relatively early. By day 9 post-infection, 100% seroconversion was observed, and peak antibody levels were recorded by day 14. Rao et al. reported similar results (37), which supports the notion that the response represents a typical immune response of sheep to SPPV infection.

PCR-based detection of viral DNA revealed that starting from day 5 post-infection, the virus was present in nearly all examined tissues. By days 11 and 14, viral DNA was no longer detectable in nasal and ocular swabs, but it persisted in whole blood and affected skin samples throughout the observation period (see Figure 6). These findings suggest that the mucosal surfaces serve as transient sites of viral replication during the acute phase, whereas blood and skin lesions support longer-term viral persistence. From a diagnostic perspective, this highlights the importance of selecting appropriate clinical material based on the time elapsed since infection. At later stages of infection, blood and skin lesions are more suitable specimens for reliable PCR-based diagnosis.

Post-mortem examination of sheep that succumbed to sheeppox revealed typical skin lesions that appeared more pronounced compared to those observed in live animals. Consistent with findings reported by Hamouda et al. (38), animals infected with the Kazakhstani isolate exhibited necrosis, thrombosis, and edema in various internal organs. However, in the present study, the severity of these lesions was notably higher, particularly in the spleen and lungs, which suggests greater virulence of the specific strain. Overall, the clinical and pathological manifestations observed in the field closely aligned with those obtained under experimental conditions.

Histological examination revealed marked pathological changes in the skin that were characteristic of the acute stage of the disease, which included massive cellular infiltration, vasculitis, and edema. Early changes were represented by perivascular infiltration, while later stages were characterized by deep destructive lesions in the dermis. Our findings are consistent with those reported by Aldaiarov et al. (39) and Abd-Elhafeiz et al. (40), who also described extensive dermal damage during sheeppox infection. Disruption of intercellular junctions, the presence of free epithelial cells within the exudate, and necrotic material extending into the mid-dermal layers were observed and were often accompanied by partial or complete loss of the epidermis. In certain areas, the formation of cavities filled with serous exudate was noted, which indicated a severe course of the cutaneous form of the disease.

Histological findings also indicated pronounced damage to pulmonary tissue, which predominantly occurred in the pleural regions. The detection of viral inclusion bodies in various cell types, particularly in fibroblasts and bronchiolar epithelium, confirms that active viral replication was occurring within these structures. The presence of epithelial hyperplasia, mitotic activity, hyperkeratosis, and apoptotic cells reflects an ongoing reparative and inflammatory tissue response to viral injury.

Examination of the lymph nodes revealed significant disruption of the tissue architecture and marked suppression of lymphoid structures. The predominance of infected epithelial-like cells and fibroblasts, along with the presence of viral inclusion bodies, indicates active viral replication within immune organs. The reduction in lymphocyte numbers coupled with a high frequency of apoptosis suggests a pronounced immunosuppressive effect exerted by the virus.

Structural alterations in the spleen were characterized by atrophy of lymphoid elements and a predominance of virus-infected cells. The high level of lymphocyte apoptosis may be attributed to the direct CPE of the virus and is likely to play a key role in the suppression of the host’s immune defense mechanisms. Thus, the obtained results indicate a high degree of viral adaptation to the Merino sheep breed and underscore the need for developing vaccination strategies that consider regional viral strains. The use of locally isolated strains in vaccine production may enhance the effectiveness of disease prevention and control efforts in endemic regions of Kazakhstan.

## 5 Conclusion

This study on the pathogenicity of a Kazakhstani SPPV isolate revealed unique characteristics that distinguish it from strains circulating in other countries. The findings indicate a high level of aggressiveness and virulence of this isolate, as evidenced by the earlier onset of clinical symptoms and high viral loads, particularly in the skin and lungs. Rapid seroconversion and severe pathological changes in internal organs further support the potential threat posed by this virus to local sheep breeds.

Phylogenetic analysis of the investigated isolate demonstrated a high degree of nucleotide identity (99.94%) with isolates identified in the Russian Federation, suggesting a close genetic relationship and potential cross-border transmission dynamics.

These results highlight the urgent need to strengthen preventive and control measures that are tailored to the specific conditions of Kazakhstan.

## Data Availability

The datasets presented in this study can be found in online repositories. The names of the repository/repositories and accession number(s) can be found at: https://www.ncbi.nlm.nih.gov/genbank/, PV434148.
